# Carpal tunnel syndrome caused by tophi in the superficial flexor tendon: a case report

**DOI:** 10.3389/fsurg.2023.1282202

**Published:** 2023-12-14

**Authors:** Haihu Hao, Weijie Kong, Han Li

**Affiliations:** ^1^ Department of Orthopedics, Shanxi Bethune Hospital, Shanxi Academy of Medical Sciences, Tongji Shanxi Hospital, Taiyuan, Shanxi, China; ^2^ Department of Orthopedics, Third Hospital of Shanxi Medical University, Taiyuan, Shanxi, China

**Keywords:** carpal tunnel syndrome, tophaceous gout, flexor tendon, surgery, case report

## Abstract

Carpal tunnel syndrome (CTS) is the most common disease among peripheral nerve entrapment diseases. CTS is often caused by the hyperplasia of the transverse carpal ligament and edema of tissue in the carpal tunnel, resulting in compression of the median nerve. Specific manifestations of CTS include numbness, loss of skin sensation in the palm and three and a half fingers on the radial side, and decreased muscle strength; however, CTS caused by wrist tophi is very rare. To our knowledge, CTS with median nerve compression caused by tophi in the superficial flexor tendon of the index finger of the wrist has not been reported before. Here, we will report a case of CTS caused by tophi in the wrist in a 37-year-old patient with no history of gout. CTS caused by tophi is uncommon, but if the patient has high uric acid, CTS may be due to tophi.

## Introduction

Carpal tunnel syndrome (CTS) is the most common clinical peripheral nerve entrapment syndrome, with the highest incidence among all peripheral nerve entrapment syndromes, usually due to the hyperplasia of the transverse carpal ligament causing compression of the median nerve (1). The disease is common in those who overuse the wrist, most of which are spontaneous, and mild patients with a short course of the disease can mostly relieve symptoms after conservative treatment (2); however, it may also be secondary to fractures and fracture dislocations about the wrist, rheumatoid arthritis, diabetes, ganglion cyst, and dialysis patients (3–6). In addition, tophi deposition in the flexor tendon sheath can also cause compression of the median nerve, leading to CTS. These conditions are uncommon and most of the cases are caused by gouty tophi in the common flexor tendon sheath of the wrist (7, 8). To our knowledge, this is the first report with pictures of tophi in the superficial flexor tendon sheath of the index finger.

Here, we present such a case of non-traumatic CTS secondary to gout and analyze the diagnosis and treatment of this disease in a 37-year-old male, who had no signs and family history of gout.

## Case report

A 37-year-old male patient came to the orthopedic outpatient clinic. His right hand showed typical CTS, that is, the numbness in the skin of three and a half fingers on the radial side of the right hand. The symptoms of finger numbness lasted for more than 6 months. The patient took non-steroidal anti-inflammatory drugs (NSAIDs), Aescuven Forte, and mecobalamin a month ago, but the symptoms were not significantly relieved. On local physical examination, there was no mass, no tenderness, no redness of the hands, and other symptoms in the wrist, and no apparent atrophy of the thenar muscle was found but Phalen's test was positive. Neurologic examination revealed numbness in the thumb, index, middle, and half ring fingers. Electromyography showed decreased conduction velocity and sensory function of the median nerve at the wrist. This patient has a normal BMI, usually smokes, drinks a small amount of alcohol, eats fewer fruits and vegetables daily, and exercises irregularly. He had no history of gout, but the uric acid level rose to 488.5 μmol/L during the examination; then, he was diagnosed with hyperuricemia.

**Figure 1:**
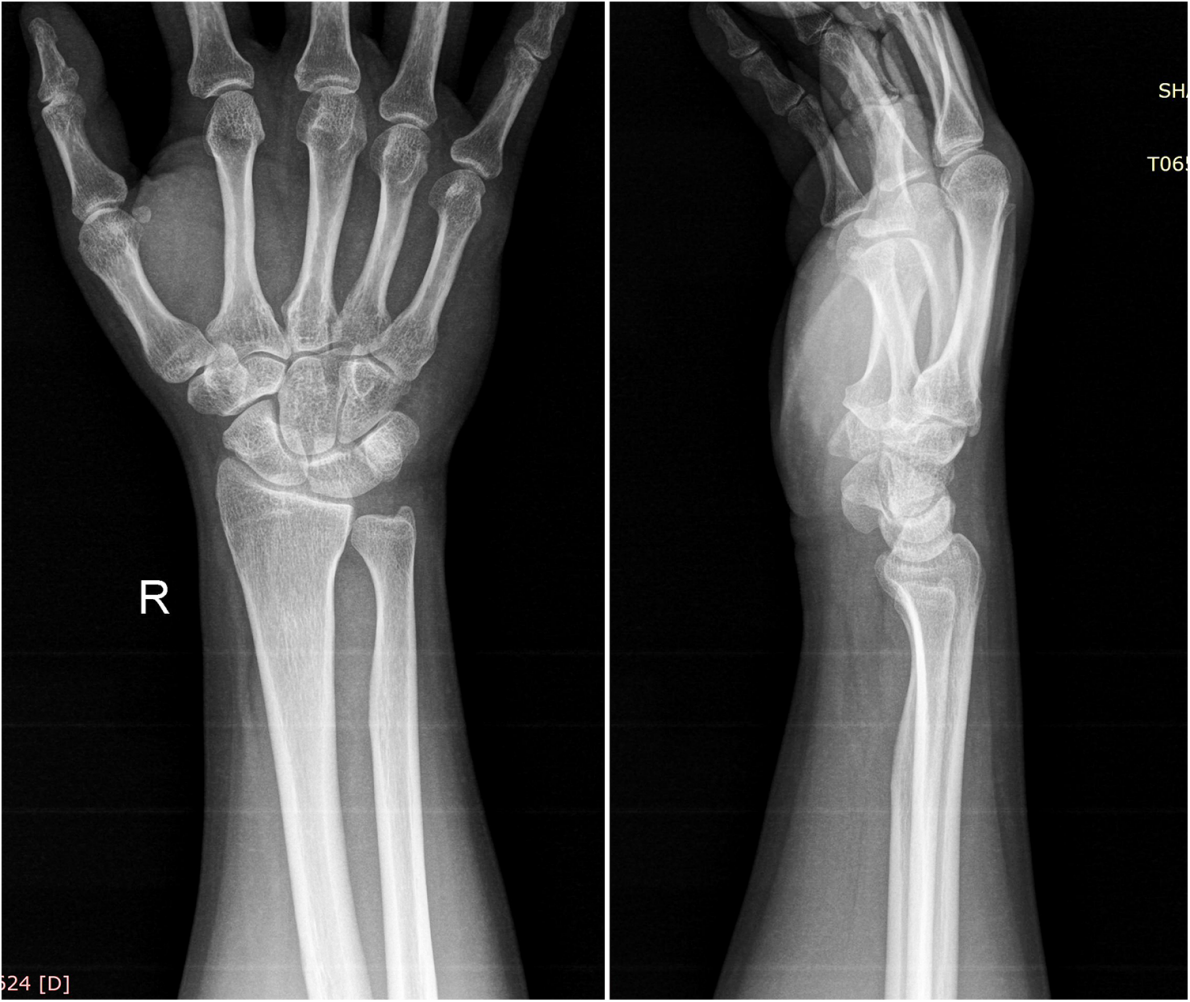
Radiology showed that no obvious abnormalities were found in the bones and soft tissues of the wrist.

**Figure 2:**
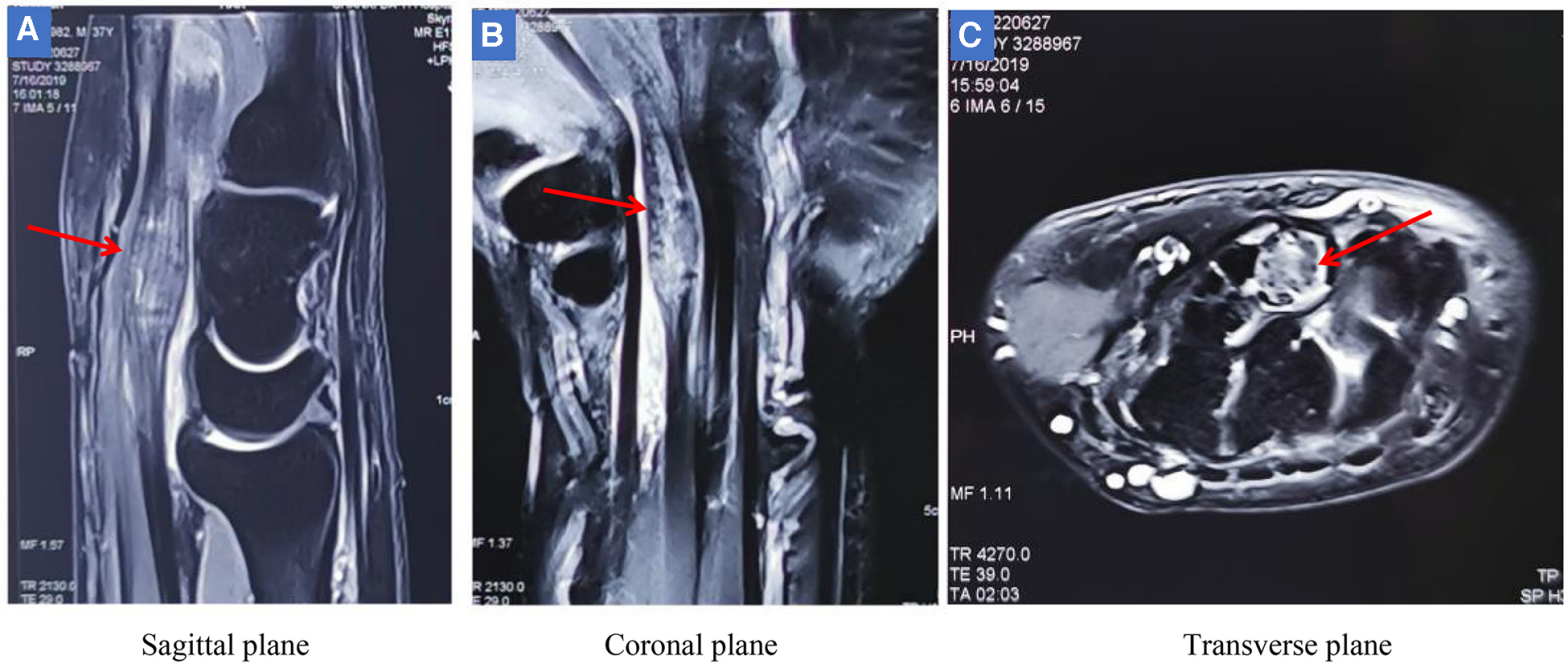
MRI showed that there was a fusiform mass on the palm side of the carpal bone (red arrow). (**A-B**) The mass was located under the transverse carpal ligament and in front of the carpal bone, and was in the shape of a pike, measuring approximately 0.66 cm 1.37 cm. (**C**) The mass can be seen in cross section compressing the median nerve. Given the patient's high uric acid levels and MRI findings, it was hypothesized that the mass could be tophi causing carpal tunnel syndrome.

Radiographs showed no tophi deposition in the bone at his wrist and hands (Figure 1). Magnetic resonance imaging (MRI) showed a fusiform mass on the volar surface of the carpal bone, and this mass happens to be pressing on the median nerve (Figure 2). Combining with the patient's x-ray, MRI, high uric acid level, and clinical manifestations, we speculate that CTS in this case was caused by tophi.

**Figure 3:**
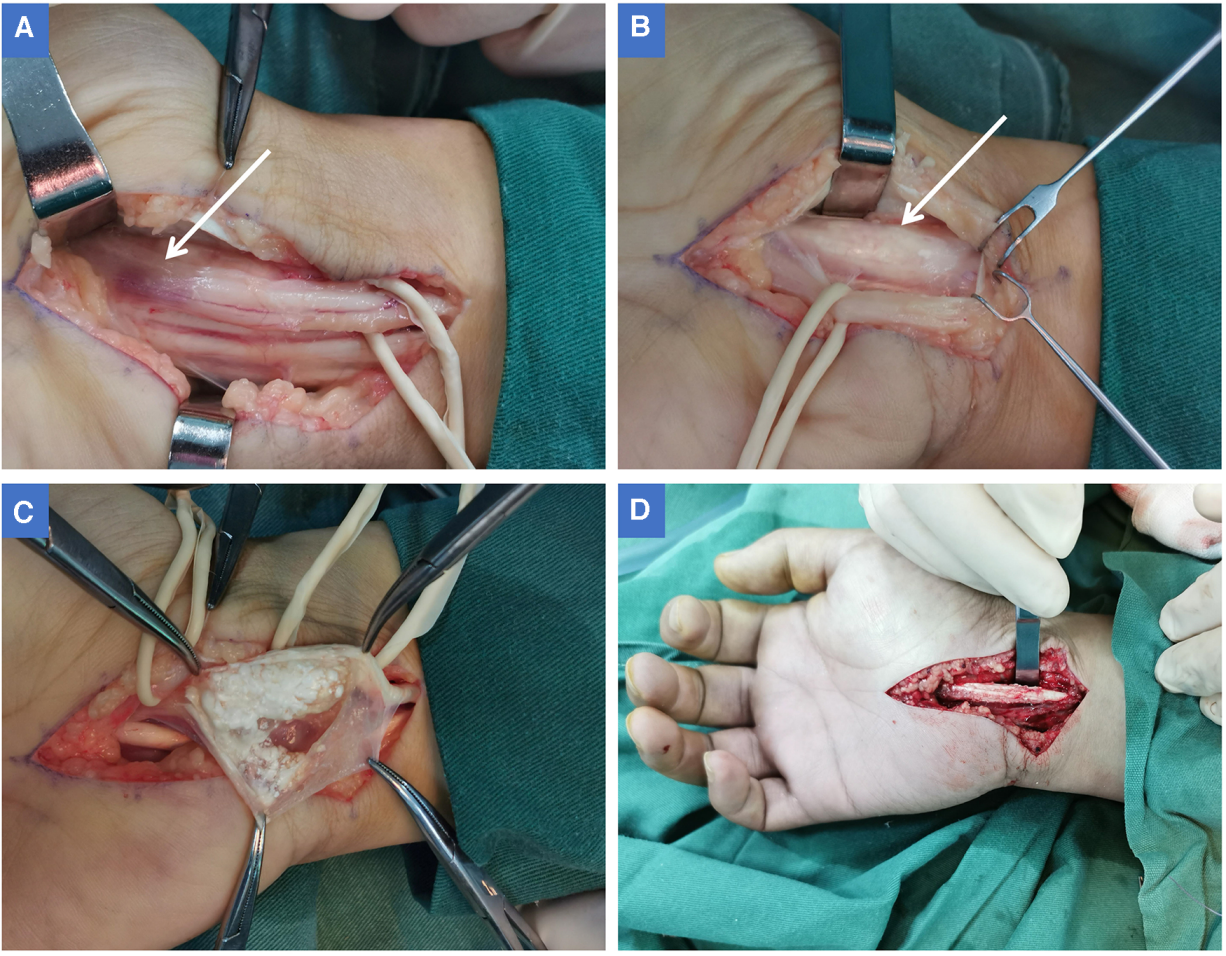
(**A**) After cutting the transverse carpal ligament, the median nerve was fully exposed. At the point where the median nerve is far from the wrist (in the palm of the hand), there is a distinct impression on the nerve, and the local color of the nerve is dark red (white arrow). (**B)** The median nerve was retracted to the ulnar side to fully expose the superficial flexor of the index finger. At this time, the flexor tendon sheath was enlarged in a fusiform shape (white arrow), and the enlarged part caused compression on the median nerve. (**C**) When the flexor tendon sheath of the index finger was opened, it can be seen that there are large number of milky white soft tophi in the tendon sheath. The tophi are entirely surrounded by the tendon sheath, and there is no inflammation or ulceration on the surface of the tendon sheath. (**D**) The tophi were removed from the tendon sheath and tenosynovectomy was performed.

In view of the fact that the patient's conservative treatment was not effective and the imaging showing that the mass was compressing the median nerve, the patient underwent surgery immediately after being hospitalized. A pneumatic tourniquet was applied to the right upper limb with a pressure of 250 mmHg, and a longitudinal incision was made on the palm side of the wrist. The transverse carpal ligament was incised to fully expose the median nerve. An indentation at the distal end of the median nerve could be seen, and the local color was dark red (Figure 3A). The median nerve was retracted to the ulnar side with a rubber strip, and the tendon sheath of the superficial flexor of the index finger was observed to be significantly swollen, squeezing the median nerve (Figure 3B). After the tendon sheath of the superficial flexor was cut, a large number of white tophi deposits were found in the tendon sheath. The surface of the tophi was soft and delicate (Figure 3C). Intraoperatively, tophi were flushed with large amounts of normal saline until there were no visible tophus deposits around the superficial flexor tendon (Figure 3D). At this time, the compression of the median nerve by the dilated flexor tendon was relieved, and the wound was flushed with saline until a clear discharge was observed. After adventitial neurolysis of the median nerve, drainage skin grafts were placed on the wound and removed the next day. It was clearly diagnosed as tophaceous gout through the pathological report on the third day after the operation.

On the second day after the operation, the patient reported that the numbness of the right thumb, index finger, middle finger, and half of the ring finger was significantly relieved.

The patient was encouraged to perform active finger flexion and extension exercises and was advised to avoid a high-purine diet and to take 40 mg of benzbromarone, a uric acid–lowering drug, orally every day. After 1 month, the dose of benzbromarone was adjusted according to the uric acid level, and the maintenance dose was generally 20–40 mg orally daily. Six months after the operation, numbness in the fingers of the patient's right hand disappeared, sensory and motor functions of the right hand fully were completely recovered, and uric acid levels were normal. As of the 12-month follow-up, the patient reported no gout attacks and no recurrence of numbness.

## Discussion

The carpal tunnel is located in the anterior region of the wrist, with the carpal bones forming the sides and bottom and the flexor retinaculum forming the top. There are the median nerve, flexor digitorum superficialis, and deep flexor digitorum tendons in the carpal tunnel; any factors that lead to narrow carpal tunnel space can cause nerve compression, such as thickening of the flexor retinaculum and swelling of tendons.

Gouty carpal tunnel syndrome has been reported relatively rarely because tophi are not a common cause of CTS. In recent years, with changes in the diet structure, the incidence of gout has been increasing, and everyone has gradually gained a new understanding about the complications of gout (9). Based on this understanding, misdiagnosis and missed diagnosis of the disease can be avoided by taking detailed medical history, combined with relevant imaging examinations and physical examination. In this case, the superficial flexor tendon of the single digit of the patient was affected by tophi, which caused the tendon to thicken locally and become fusiform. To the best of our knowledge, tophi deposits in the superficial flexor tendon of a single finger have never been reported in the literature. There are many early manifestations of gout, and it is often pain in the first metatarsophalangeal (MTP) joint, but the hands are not common presenting locations (10). Because carpal tunnel compression is so rare as an early manifestation of gout, it is often overlooked in diagnosis.

In this case, it is particularly extraordinary because the patient has no family history of gout, and there is no gout manifestation in other parts of the body. Only MRI examination and the patient's uric acid level suggest that tophi may be the possible cause of CTS. If left undiagnosed, irreversible nerve damage and complications may occur, including joint changes, tendon rupture, and tenosynovitis (11). Therefore, when considering the causes of CTS, all possibilities must be considered.

For the wrist, MRI is ideally suited for the suspected soft tissue disease because of its multiplanar capability and excellent soft tissue contrast. In this case, we unexpectedly discovered a mass forming in the flexor tendon of the wrist with MRI, which is essential for presurgical planning to assess the location and size of the soft lesion. In addition, imaging examinations such as ultrasound and CT are recommended for early and accurate diagnosis (12, 13), but CT is mainly used to display the bone structures. Various examination methods have their advantages and disadvantages. Sometimes we need to combine multiple examinations to obtain a clear diagnosis.

Poor wound healing may occur due to the inflammatory nature of gout, but no wound healing problems were encountered in this case. This may be related to the best efforts to remove tophi during the operation and the continuous flushing of tophi with normal saline. Residues of tophi are unavoidable, although thorough cleaning and flushing have been achieved during the operation, so the disease may recur. It is necessary to use uric acid–lowering drugs, reduce purine-rich meat, and maintain serum uric acid levels to normal after surgery to avoid gout attacks. In this case, the tophus may be removed more thoroughly and uric acid–lowering drugs are taken regularly after the operation, so there is no recurrence so far, and good results have been achieved.

In short, it is necessary to combine ultrasound, MRI, and other examinations to determine the diagnosis and the specific etiology of CTS. In order to avoid permanent damage to the median nerve due to compression, it is recommended to perform surgery to decompress and altogether remove tophi completely and to control treatment with diet and uric acid–lowering drugs after surgery.

## Data Availability

The raw data supporting the conclusions of this article will be made available by the authors, without undue reservation.
