# Supplementary material for: Carpal tunnel syndrome caused by tophi in the superficial flexor tendon: a case report
Source: Front Surg. 2023 Dec 14;10:1282202. doi: 10.3389/fsurg.2023.1282202 (PMC10757969; doi:10.3389/fsurg.2023.1282202)

Supplementary Material

1. The patient's preoperative EMG suggested that the median nerve at the right wrist was consistent with electrophysiological findings of severe damage.

Diagnosis comments:

1) EMG: A small amount of spontaneous potentials were seen in the muscle portion examined in the upper limb, and the recruitment reactions were in simple phase, simple-mixed phase, and mixed phase respectively.

(2) NCV: The MCV of the right median nerve was not measured, and the MCV of the remaining ulnar nerve and radial nerve were within the normal range, and the latency of the innervating muscle CMAP endings was normal, with normal wave amplitude; the SCV of the right median nerve was not detected, but the SCV of the rest of the examined ulnar nerve and superficial branch of the radial nerve was in the normal range, and the SNAP latency was normal, with normal amplitude of the wave work..

(3) F-wave: the F-wave of the right ulnar nerve is good, and the latency is within the normal range.

EMG conclusion: The median nerve at the right wrist was consistent with electrophysiological findings of severe damage.

ID #:05637

山西医学科学院 山西大医院

肌电图&诱发电位检查报告

姓 名: 杨健 性 别: 男 年 龄: 37 岁

病 区: 骨科一病区 电 话: ID 号: 05637

登记号: 身高/体重: 日 期: 2019-7-15 12:17

Motor Nerve Conduction（运动传导）:

神经及刺激点	潜伏期 ms	波幅 mV	分段	潜伏期差 ms	距离 mm	传导速度 m/s
Median.R						
Wrist	/	/	APB-Wrist	/	/	/
Elbow	/	/	Wrist-Elbow	/	/	/

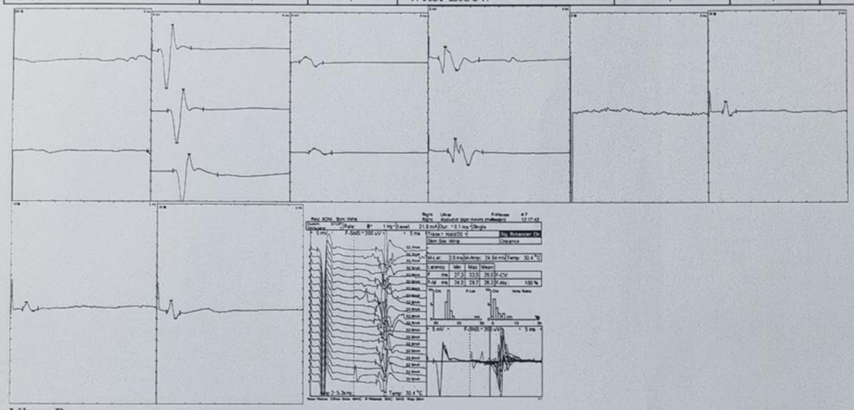

神经及刺激点	潜伏期 ms	波幅 mV	分段	潜伏期差 ms	距离 mm	传导速度 m/s
Ulnar.R						
Wrist	2.4	31.93	Abductor digiti minimi (manus)-Wrist	2.4		
Below elbow	6.1	26.68	Wrist-Below elbow	3.7	200	54.0
Above elbow	8.1	24.94	Below elbow-Above elbow	2.0	100	50.0
Radial.R						
Forearm	3.4	4.622	Extensor indicis proprius-Forearm	3.4		
Lateral brachium	6.8	2.786	Forearm-Lateral brachium	3.4	170	50.0
Median.L						
Wrist	3.7	11.43	APB-Wrist	3.7		
Elbow	7.0	12.82	Wrist-Elbow	3.3	200	60.6

Sensory Nerve Conduction（感觉传导）:

神经及刺激点	潜伏期 ms	波幅μV	分段	潜伏期差 ms	距离 mm	传导速度 m/s
Median.R						
Middle palm	/	/	Digit II (index finger)-Middle palm	/	/	/
Median.L						
Middle palm	2.1	27.81	Digit II (index finger)-Middle palm	2.1	145	69.0
Ulnar.R						
Wrist	1.7	8.084	Digit V (little finger)-Wrist	1.7	120	70.5

Radial.R

Forearm	1.4	34.72	Anatomical snuff box-Forearm	1.4	90	64.2

F-Wave Studies（F 波）

神经	M 波潜伏期	F 波平均潜伏期	F 波出现率(%)
Ulnar.R	2.8	29.0	100.0

Needle EMG Examination:

Muscle	Insertional	Spontaneous Activity			Volitional MUAPs			Max-Voliti
	Insertional	Fibs	+ Wave	Fasc	Dur(ms)	Amp(uv)	Ploy	
Abductor pollicis brevis.R	Normal	1+	2+	None	8.3	119	0.0	
Abductor pollicis brevis.L	Normal	None	None	None	10.3	1112	0.0	
Abductor digiti minimi (manus).R	Normal	None	None	None	12.5	305	0.0	
Abductor digiti minimi (manus).L	Normal	None	None	None	12.6	194	0.0	
Extensor indicis proprius.R	Normal	None	None	None	7.0	188	0.0	

诊断意见：

（1）EMG：上肢所检肌部分见少量自发电位，募集反应分别呈单纯相，单纯-混合相，混合相；（2）NCV：右侧正中神经 MCV 未测出，其余所检尺神经、正中神经、桡神经 MCV 在正常范围，支配肌 CMAP 末梢潜伏期正常，波幅正常；右侧正中神经 SCV 未测出，其余所检尺神经、桡浅神经 SCV 在正常范围，SNAP 潜伏期正常，波幅正常；（3）F 波：右侧尺神经 F 波出波好，潜伏期在正常范围。

肌电图提示：右侧正中神经（腕部）重度损害电生理表现，请结合临床。

医师签名：

（本报告仅供临床参考，不作为诊断依据） 2019-7-15

2. The picture of the numb areas of the hands is provided. The patient had paraesthesiae in the three and a half radial fingers of his right hands.

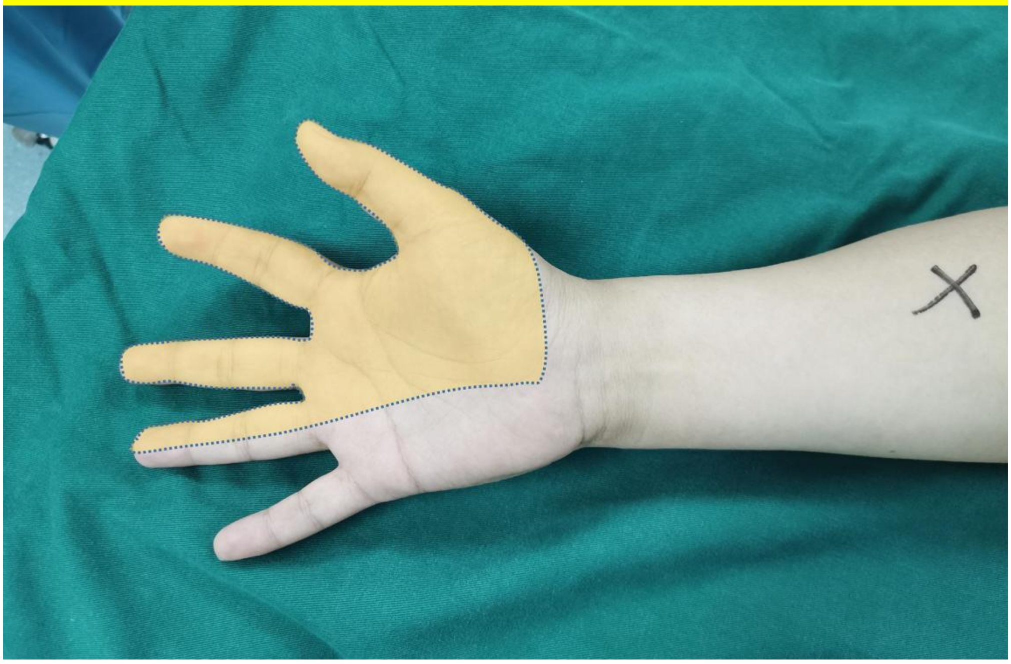

Supplement: Supplementary file 1 [file Presentation1.pdf]
